# Adjusting Breast Cancer Patient Prognosis with Non-HER2-Gene Patterns on Chromosome 17

**DOI:** 10.1371/journal.pone.0103707

**Published:** 2014-08-06

**Authors:** Vassiliki Kotoula, Mattheos Bobos, Zoi Alexopoulou, Christos Papadimitriou, Kyriaki Papadopoulou, Elpida Charalambous, Eleftheria Tsolaki, Grigorios Xepapadakis, Irene Nicolaou, Irene Papaspirou, Gerasimos Aravantinos, Christos Christodoulou, Ioannis Efstratiou, Helen Gogas, George Fountzilas

**Affiliations:** 1 Department of Pathology, Aristotle University of Thessaloniki School of Medicine, Thessaloniki, Greece; 2 Laboratory of Molecular Oncology, Hellenic Foundation for Cancer Research, Aristotle University of Thessaloniki School of Medicine, Thessaloniki, Greece; 3 Department of Biostatistics, Health Data Specialists Ltd, Athens, Greece; 4 Department of Clinical Therapeutics, “Alexandra” Hospital, University of Athens School of Medicine, Athens, Greece; 5 Breast Clinic, “REA” Hospital, Piraeus, Greece; 6 Department of Histopathology, “Agii Anagriri” Cancer Hospital, Athens, Greece; 7 Department of Pathology, “Alexandra” Hospital, Athens, Greece; 8 Second Department of Medical Oncology, “Agii Anargiri” Cancer Hospital, Athens, Greece; 9 Second Department of Medical Oncology, “Metropolitan” Hospital, Piraeus, Greece; 10 Department of Pathology, “Papageorgiou” Hospital, Thessaloniki, Greece; 11 First Department of Medicine, “Laiko” General Hospital, University of Athens, Medical School, Athens, Greece; 12 Department of Medical Oncology, “Papageorgiou” Hospital, Aristotle University of Thessaloniki School of Medicine, Thessaloniki, Greece; University of Torino, Italy

## Abstract

**Background:**

*HER2* and *TOP2A* gene status are assessed for diagnostic and research purposes in breast cancer with fluorescence in situ hybridization (FISH). However, FISH probes do not target only the annotated gene, while chromosome 17 (chr17) is among the most unstable chromosomes in breast cancer. Here we asked whether the status of specifically targeted genes on chr17 might help in refining prognosis of early high-risk breast cancer patients.

**Methods:**

Copy numbers (CN) for 14 genes on chr17, 4 of which were within and 10 outside the core *HER2* amplicon (HER2- and non-HER2-genes, respectively) were assessed with qPCR in 485 paraffin-embedded tumor tissue samples from breast cancer patients treated with adjuvant chemotherapy in the frame of two randomized phase III trials.

**Principal Findings:**

*HER2*-genes CN strongly correlated to each other (Spearman’s rho >0.6) and were concordant with FISH *HER2* status (Kappa 0.6697 for *ERBB2* CN). *TOP2A* CN were not concordant with *TOP2A* FISH status (Kappa 0.1154). CN hierarchical clustering revealed distinct patterns of gains, losses and complex alterations in HER2- and non-HER2-genes associated with IHC4 breast cancer subtypes. Upon multivariate analysis, non-HER2-gene gains independently predicted for shorter disease-free survival (DFS) and overall survival (OS) in patients with triple-negative cancer, as compared to luminal and HER2-positive tumors (interaction p = 0.007 for DFS and p = 0.011 for OS). Similarly, non-HER2-gene gains were associated with worse prognosis in patients who had undergone breast-conserving surgery as compared to modified radical mastectomy (p = 0.004 for both DFS and OS). Non-HER2-gene losses were unfavorable prognosticators in patients with 1–3 metastatic nodes, as compared to those with 4 or more nodes (p = 0.017 for DFS and p = 0.001 for OS).

**Conclusions:**

*TOP2A* FISH and qPCR may not identify the same pathology on chr17q. Non-HER2 chr17 CN patterns may further predict outcome in breast cancer patients with known favorable and unfavorable prognosis.

## Introduction

Chromosomal instability (CIN), defined as losses or gains of multiple chromosomal areas [1], represents one aspect of genome instability that underlies all hallmarks of cancer [2]. Within CIN, acquisition of increased numbers of gene copies, i.e. gene amplification, is a common event. For routine diagnostic and research purposes employing formalin-fixed paraffin-embedded tissues (FFPE), gene copies are usually evaluated with fluorescence in situ hybridization (FISH) on interphase chromosomes in truncated nuclei. In breast cancer in particular, FISH assays for the assessment of *HER2 (ERBB2)* gene status on chromosome (chr) 17q12 are used as in vitro diagnostic devices (IVD), based on the well established clinical utility of this marker for selecting patients who will benefit from trastuzumab treatment. Typically, dual FISH assays probing the *HER2* gene and the chr17 centromeric regions (CEN17) are used for diagnostics, under strict interpretation guidelines [3]. Triple assays, detecting CEN17, *HER2* and *TOP2A*, a gene on 17q21, have also received IVD license. However, as shown with methods evaluating larger parts of, or entire chromosomes, an increased number of *HER2* gene copies on 17q12 may occur as a single event on an otherwise stable chr17 or it may accompany a broad spectrum of changes on the same chromosome. In addition, chr17 may be unstable without increased *HER2* copies [4]–[8], or chr17 may not be intact [5], [9]. The above aspects of chr17 instability may be relevant to breast cancer patient outcome, at various disease stages and treatment settings, but are missed with the currently used FISH assays and their interpretation, as shown with the use of distinct evaluation of CEN17 signals [10] and application of multiple FISH assays for chr17 [11].

Another concern with the currently used bacterial artificial chromosome probes for FISH is that they span large chromosomal areas; for example, 5 Mb for the CEN17, 600 Kb for the HER2 and 500 Kb for the TOP2A probes in the triple assay. Given that the targeted genes are 53 Kb (*ERBB2*) and 38 Kb (*TOP2A*), and that the distance between them and their neighboring genes is occasionally short, FISH probes in fact cover a multitude of genes in addition to the annotated one. Further, these probes need to be cut in pieces of maximally 500 bp for efficient labeling and hybridization. Since 10 Kb fluorescent signals can be detected with most modern image analysis systems, the case may be that the probes in fact detect fragments of the targeted regions, which do not necessarily include the annotated genes.

In the present study we used qPCR for the assessment of somatic copy number (CN) alterations in 14 genes on chr17p and chr17q, including *ERBB2* and *TOP2A*. CN alterations were compared to those assessed by classic FISH and were evaluated for their impact on the outcome of patients with operable high-risk breast cancer treated with anthracycline-containing regimens in the pre-trastuzumab era.

## Materials and Methods

The study was performed on FFPE tissues from a series of tumors derived from patients with operable high-risk breast cancer who had been treated within the frame of two randomized phase III trials by the Hellenic Cooperative Oncology Group (HeCOG), HE10/97 [12] and HE10/00 [13], [14]. Patients had undergone modified radical mastectomy (MRM) or breast-conserving surgery (BCS) and had received adjuvant E-T-CMF except for the control arm in HE10/97 who had received E-CMF only. Patients with HER2-positive tumors had received trastuzumab upon relapse. Clinical protocols were approved by local regulatory authorities and were also included in the Australian New Zealand Clinical Trials Registry (ANZCTR) and allocated the following Registration Numbers: ACTRN12611000506998 (HE10/97) and ACTRN12609001036202 (HE10/00). The present translational research protocol was approved by the Bioethics Committee of the Aristotle University of Thessaloniki School of Medicine under the general title “Molecular investigation of the predictive and/or prognostic role of important signal transduction pathways in breast cancer” (A7150/18-3-2008). All patients signed a study-specific written informed consent before randomization, which in addition to giving consent for the trial allowed the use of their biological material for future research purposes. Tumors had previously been subtyped with IHC4 and FISH on tissue microarrays (TMA), including two 1.5 mm cores per tumor [15]. Data for *TOP2A* gene status were also available [16].

For easily distinguishing between parameters assessed with different methods, throughout this text, HER2 refers to the HER2 amplicon, to results obtained by FISH and array-based comparative genomic hybridization (aCGH), to tumor HER2 status and to breast cancer subtypes, while *ERBB2* is used for results obtained specifically for sequences of this gene, e.g. with qPCR methods.

### Immunohistochemistry (IHC) and triple FISH

IHC protocols for the ER, PgR, HER2 and Ki67 data used in this study have previously been described [15]. Briefly, HER2 was scored in a 4-scale from 0–3, with intense membrane staining in >30% invasive tumor cells classified as positive (3+ staining) [17]. Cut-offs were set for ER and PgR at 1% positive nuclei [18], and for Ki67 at 14% [19]. For the purposes of the present study, ER and PgR simultaneous staining was considered as one parameter (hormone receptor status, HRS). Ki67 was evaluated as a continuous variable (% of positively stained nuclei); the highest score for each TMA core from the same tumor was recorded. FISH was evaluated in twenty tumor nuclei [20]. The *HER2* gene was classified as amplified for *HER2*/CEN17 ratios ≥2.2 [17], or for mean *HER2* copy numbers >6 [21]. The *TOP2A*/CEN17 ratio cut-off for *TOP2A* amplification was ≥2.0 [22].

### Chr17 CN with qPCR

**Figure 1:**
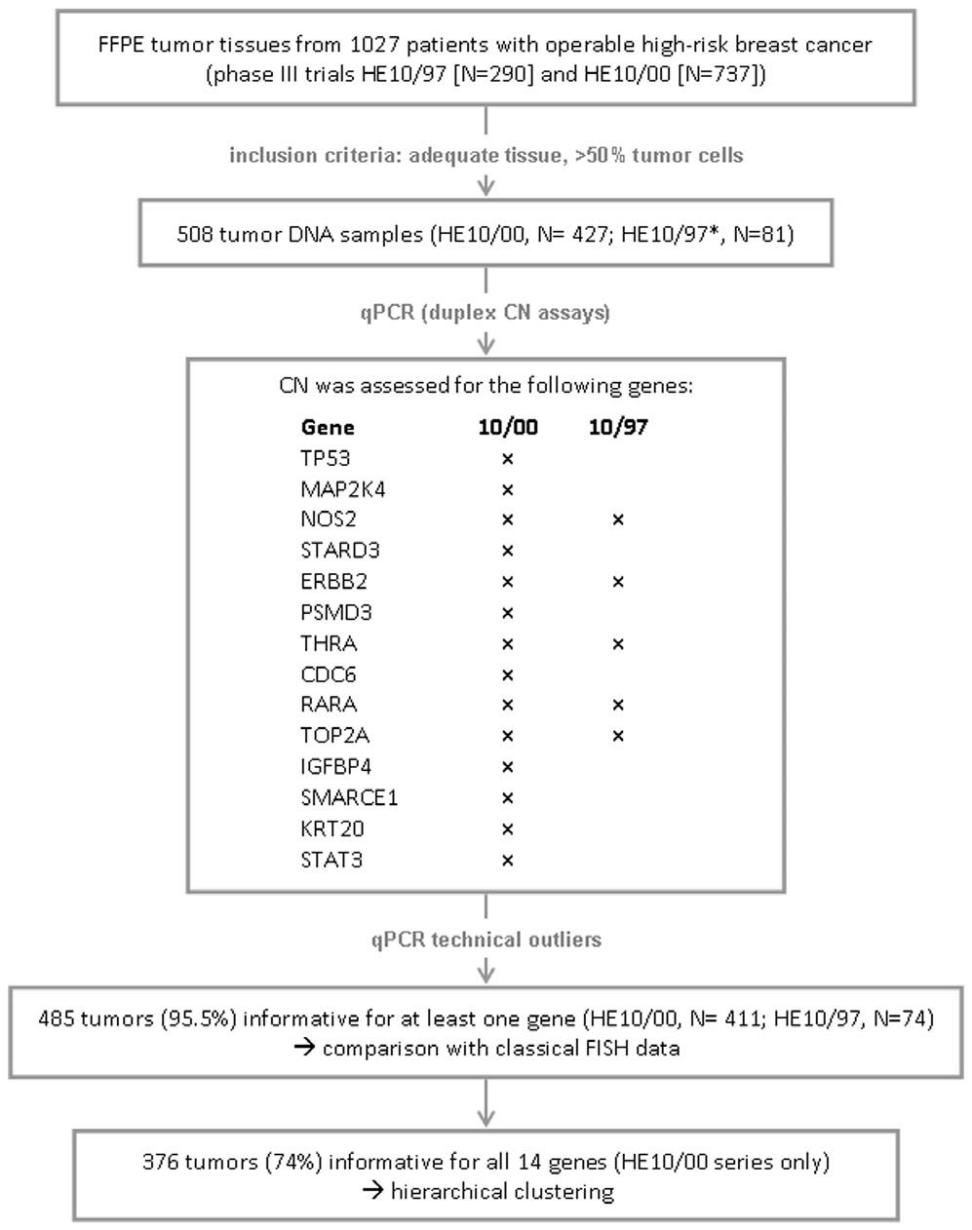
REMARK flow chart. *: for the majority of HE10/97 cases tissue material was exhausted while for the 81 cases from the same series the amount of available DNA was sufficient for a limited number of genes only.

To be able to directly compare classic FISH and qPCR results, DNA was extracted from TMA cores instead of whole tumor sections. DNA was extracted manually with the VERSANT Tissue Preparation Reagents kit (Siemens Healthcare, Erlangen, Germany) [23]. Only cases represented with >50% tumor cells on core sections, as determined by a certified pathologist (M.B.), were included in this study (**Figure 1**). With this restriction, of the originally 1027 tumors that had been processed on TMAs from the HE10/00 and HE10/97 series, 508 cases were eligible for DNA extraction, 427 from the HE10/00 and 81 from the HE10/97 trial. CN was assessed for *TP53, MAP2K4, NOS2, STARD3, ERBB2, PSMD3, THRA, CDC6, RARA, TOP2A, IGFBP4, SMARCE1, KRT20* and *STAT3* (14-gene set), spanning 17p13.1–17q21.31, with premade CNV assays (Life Technologies/Applied Biosystems). Official gene names are according to http://www.ncbi.nlm.nih.gov/gene/. Due to the limited quantity of HE10/97 DNA samples, only *NOS2, ERBB2, THRA, RARA* and *TOP2A* (5-gene set) were amplified for this series. For each gene, two intron-exon overlapping genomic targets, located proximally (5′) and distally (3′) to the promoter, were amplified, in order to obtain information on the entire gene length. The method involves duplex reactions, including for the target gene, FAM labeled TaqMan minor groove binding (MGB) probes and for the reference gene, Taqman VIC -TAMRA labeled probes, both assays with unlimited primers. Assay IDs are shown in **Table S1**. TaqMan Copy Number Reference Assay RNase P was used as endogenous reference. Reactions (10 ul, 10 ng template per reaction) were run in quadruplicates in an ABI7900HT system, in 384-well plates under default conditions (45 cycles of amplification; reading threshold at 0.1). Five peripheral blood DNA samples from non-cancer patients were included in each run as calibrator samples, along with no-template controls (NTC). Results were obtained automatically with the CopyCaller Software v2.0, as predicted copy numbers in comparison to averaged calibrator values, upon setting the evaluation threshold at CT = 33 for reference RNase P in each reaction. Samples with reference CT>33 were excluded from analysis. Z-scores for all accepted samples and CN range for replicates were <1. No amplification curves were observed for NTCs.

### Evaluation of qPCR CN

CopyCaller predicted CN for each gene were initially evaluated separately for the 5′ and 3′ amplicons (**Table S2**) and analyzed as (a) categorical and (b) continuous variables.

For (a), CN were classified into a 5-scale variable as follows: loss: 0.5–1 copies; normal: 1–2 copies; marginal: 3–4 copies; low gains: 5–6 copies; high gains: >6 copies. Because of significant differences in the obtained classification for the 5′ and 3′ ends for some genes, gene status was finally classified according to maximal CN. This compromise was necessary in order to obtain one single status per gene; it was preferred over getting the average of two values for amplicons located distantly to each other in long genes, since structural aberrations cannot be tested with this method. The number of informative cases per gene amplicon and the final number of tumors eligible for single gene analysis is shown in **Figure S1**. Out of 508 tumors from HE10/00 and HE10/97, results for at least one gene were obtained in 485 cases (95.5%), 411 of which were derived from the HE10/00 series.

For (b), CN values were transformed with the natural logarithm (neper logarithm = 2.78). Log transformation was considered necessary because of the broad range and right skewness of the CN values observed for some genes (**Figure S1, Table S1**); the natural log was preferred because it provides values closer to median. The distribution of these values is shown in **Figure S2**. Again, for log transformation, single gene status as described above, as well as 5′ and 3′ amplicon CN were used. Log transformed CN values were subjected to unsupervised hierarchical clustering with the JMP v.8 software (SAS Institute Inc., Cary, NC), using the Ward’s minimum variance method. The clusters identified with this approach were used as categorical variables for comparisons with clinicopathological variables and patient outcome. Clustering with the 14-gene set was informative in 376 of 416 HE10/00 samples (90%).

The demographic and clinicopathological characteristics for patients with informative tumors in (a) and (b) are shown in **Table 1**.

**Table 1:** Demographic and clinicopathological characteristics of patients and tumors from HE10/00 & HE10/97 cohorts.

Parameter	Categories	5 genes∧ (HE10/00 & HE10/97)	All 14 genes (HE10/00 only)
Patients	N	485	376
Age (continuous)	Mean (SD)	53.3 (11.4)	54.3 (11.0)
	Min–Max	22–79	28–77
Age (categorical)	34–50	173 (35.6%)	127 (33.8%)
	<34	20 (4.2%)	11 (3%)
	>50	292 (60.2%)	238 (63.2%)
Menopausal Status	Post	279 (57.6%)	230 (61.2%)
	Pre	206 (42.4%)	146 (38.8%)
Study	HE10/00	411 (84.8%)	376 (100%)
	HE10/97	74 (15.2%)	
Type of surgery	MRM	315 (65%)	236 (62.8%)
	BCS	170 (35%)	140 (37.2%)
Tumor size	>2 cm	350 (72.2%)	266 (70.8%)
	≤2 cm	135 (27.8%)	110 (29.2%)
Histological grade	I–II	216 (44.6%)	169 (45%)
	III-Undifferentiated	269 (55.4%)	207 (55%)
Histological type	Mixed	27 (5.6%)	20 (5.4%)
	Comedo	11 (2.2%)	9 (2.4%)
	Medullary	10 (2%)	7 (1.8%)
	Papillary	3 (0.6%)	
	Inflammatory	6 (1.2%)	3 (0.8%)
	Invasive ductal	389 (80.2%)	306 (81.4%)
	Invasive lobular	39 (8%)	31 (8.2%)
N of positive nodes	0	1 (0.2%)	
	1 to 3	274 (56.4%)	176 (46.8%)
	>3	210 (43.2%)	200 (53.2%)
Treatment**	E-T-CMF	449 (92.6%)	376 (100%)
	E-CMF	36 (7.4%)	
Adjuvant HT	Yes	370 (78.1%)	280 (76.3%)
	No	104 (21.9%)	87 (23.7%)
Adjuvant RT	Yes	365 (77.7%)	279 (77.1%)
	No	105 (22.3%)	83 (22.9%)
ER/PgR status	Negative	127 (26.2%)	93 (24.7%)
	Positive	357 (73.8%)	283 (75.3%)
HER2	Negative	362 (75.4%)	280 (75.1%)
	Positive (3+ or ampl)	118 (24.6%)	93 (24.9%)
Ki67	High (≥14%)	338 (70.1%)	252 (67.4%)
	Low (<14%)	144 (29.9%)	122 (32.6%)
TOP2A (FISH)	Deleted	26 (5.4%)	18 (4.8%)
	Non-amplified	399 (82.2%)	311 (82.8%)
	Amplified	54 (11.2%)	43 (11.4%)
	Equivocal	6 (1.2%)	4 (1%)
TOP2A (FISH) binary	Amplified	54 (11.2%)	43 (11.4%)
	Non-amplified	431 (88.8%)	333 (88.6%)
Subtypes	Luminal A	110 (23.0%)	95 (25.5%)
	Luminal B	184 (38.5%)	139 (37.3%)
	Luminal-HER2	57 (11.9%)	46 (12.3%)
	HER2-enriched	60 (12.6%)	47 (12.6%)
	TNBC	67 (14.0%)	46 (12.3%)

*according to REMARK (Figure 1);

∧NOS2, ERBB2, THRA, RARA, TOP2A; MRM = modified radical mastectomy; BCS = breast conserving surgery;

**E = epirubicin; T = paclitaxel (Taxol); C = cyclophosphamide; M = methotrexate; F = fluorouracil.

### Statistics

Categorical data are presented as numbers and corresponding percentages, continuous data as median and range values. Fisher’s exact or Pearson’s chi-square tests were used for group comparisons of categorical data; non-parametric Spearman’s correlation was used for the evaluation of continuous CN in paired gene comparisons, whereby rho (correlation coefficient) values >0.5 were considered to be significant. With respect to continuous Ki67 IHC data, ROC-curve analysis with DFS and OS at 5-years, as clinical endpoints, did not prove helpful for setting a specific cut-off in HER2-positive and triple-negative tumors; hence, the 14% Ki67 cut-off was used for all tumors.

Concordance between *HER2* and *TOP2A* FISH and CN status was evaluated with (a) the Kappa coefficient, whereby Kappa of 1 corresponds to complete agreement, between 0.75 and 1 to strong, between 0.4 and 0.75 to fair, and, <0.4 to poor agreement; (b) the McNemar test for comparing agreement between binary FISH (negative vs. positive) and CN values (no gains vs. gains); and (c) multiple correspondence analysis (MCA) for drawing conclusions about the underlying relationship between FISH data and gene CN.

Disease-free survival (DFS) was measured from the date of diagnosis until verified disease progression, death or last contact, whichever occurred first, and overall survival (OS) from diagnosis until death from any cause or date of last contact. Time-to-event distributions were estimated using the product limit method. Kaplan-Meier curves and log-rank tests were used for comparing time to event distributions and evaluating DFS and OS differences, while univariate Cox regression analysis was used for reporting hazard ratios. Cox regression was also applied for testing interactions between the identified clusters and standard clinicopathological parameters, i.e. effects produced on outcome by one variable examined according to different levels of the other. In multivariate Cox regression analysis, cluster interactions were adjusted for significance against clinicopathological variables among the following (categories as in **Table 1**): age, treatment group, menopausal status, histological grade, tumor size, number of positive axillary nodes, Er/PgR and HER2 status, Ki67 IHC, adjuvant hormonotherapy and type of operation. The examined interactions were included in the final model, in order to investigate whether they added independent prognostic information, as compared to the significant clinicopathological parameters. In multivariate analysis, significance was determined at the level of 15% and in univariate at 5%. All tests were two-sided.

The SAS software was used for statistical analysis (SAS for Windows, version 9.3, SAS Institute Inc., Cary, NC), while no adjustments for multiple comparisons were reported. Statistical analysis complied with the reporting recommendations for tumor marker prognostic studies [24].

Raw data (gene target per run) including the overview of runs can now be found at http://hecog-images.gr/17CN/.

## Results

### Chr17 gene CN strongly correlate with each other within the core HER2 amplicon

The associations between single gene max CN (5-scale, categorical) and standard clinicopathological parameters are shown in **Table S3**. High CN (gains and amplification) were observed significantly more often in tumors from patients >50 years old for *ERBB2* (p = 0.014) and *SMARCE1* (p = 0.012); in post-menopausal patients for *NOS2* (p = 0.038), *ERBB2* (p = 0.015), *PSMD3* (p = 0.008), *IGFBP4* (p = 0.030) and *STAT3* (p = 0.011); in high-grade tumors for *STARD3* (p = 0.009), *ERBB2* (p = 0.004) and *CDC6* (p = 0.022); and, in ER/PgR-negative tumors for *STARD3* and *ERBB2* (both p<0.001). High CN for *ERBB2* and four additional genes in its proximity (*STARD3* centromerically; *PSMD3, THRA* and *CDC6* telomerically) strongly coincided with positive classic HER2 status, as expected (all p’s <0.001). High CN for the same genes coincided with amplified *TOP2A* by FISH, but unexpectedly, *RARA, TOP2A* and *IGFBP4*, i.e. the genes covered by the TOP2A FISH probe, did not.

**Figure 2:**
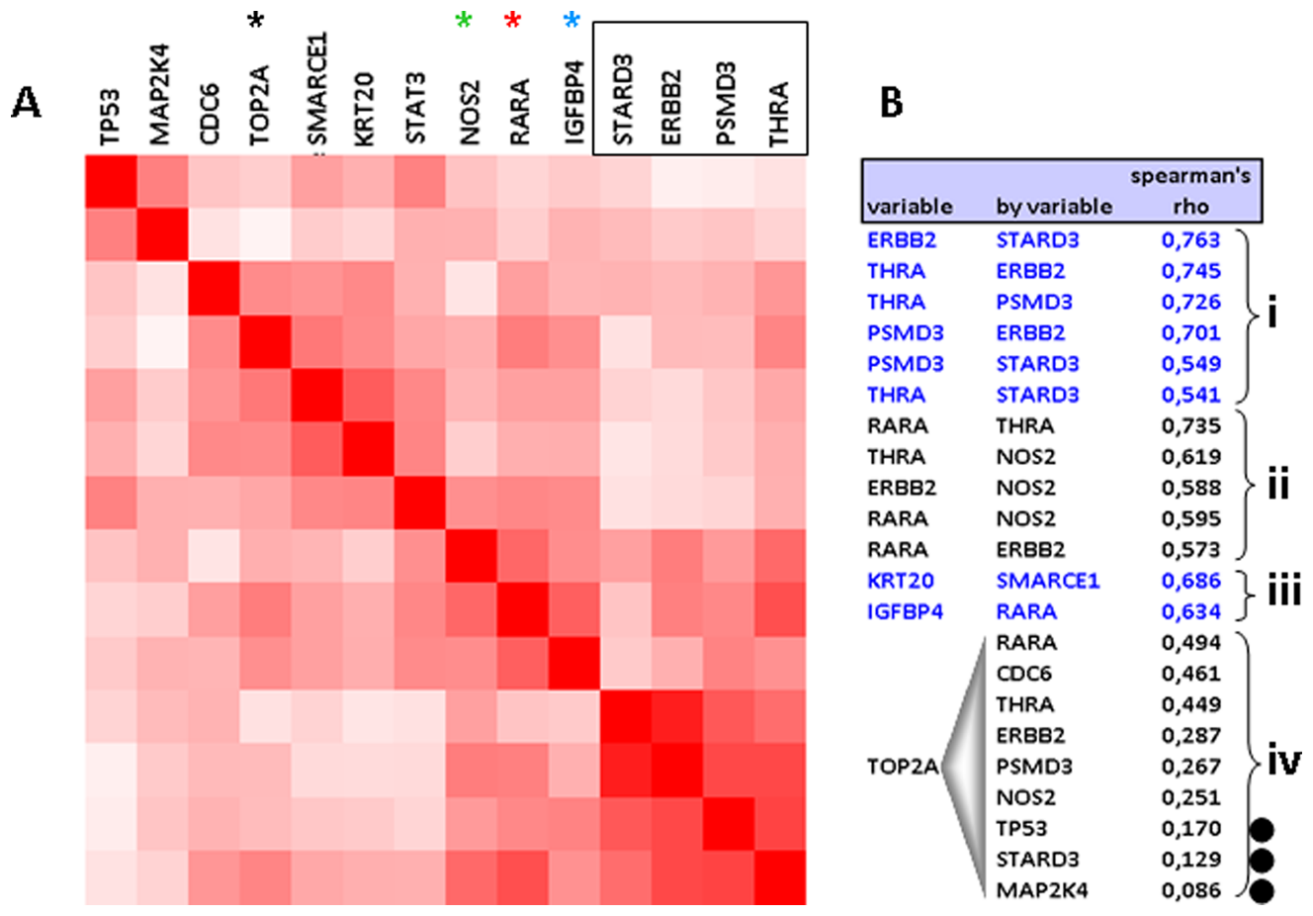
Correlations of log transformed copy number (CN) values. **A:** correlation map (clustered). **B:** Significant Spearman’s rho (**i, ii, iii**) and TOP2A correlations (**iv**). **Except for those marked with a black dot in B, all other correlations yielded p values <0.0001. However, concerning the degree of correlation (rho values),** the most significant correlations are observed for the examined genes within the core HER2 amplicon (boxed in **A**, part **i** in **B**). NOS2 (centromerically to ERBB2), as well as RARA and IGFBP4 (telomerically to THRA) correlate with the core *HER2* amplicon status (colored stars in **A**, part **ii** in **B**). Interestingly, the CN status of the *TOP2A* gene, which is located between RARA and IGFBP4, differs from the status of these two genes (black star in **A**); *TOP2A* CN are vaguely correlated to the other genes examined (part **iv** in **B**).

CN for *ERBB2* and its neighbor genes (*STARD3, PSMD3, THRA*) strongly correlated with each other with all analytical approaches, i.e., as 5′ and 3′ end results in the 5-scale classification (**Figure S3**) or as continuous log-transformed values (**Figure 2**). CN for NOS2, located centromerically to *ERBB2*, as well as for *RARA* and *IGFBP4* located telomerically to *THRA*, also strongly correlated to the four HER2-amplicon-related genes and to each other. By contrast, CN for the TOP2A gene, which is located between *RARA* and *IGFBP4*, vaguely or minimally correlated with these two and with HER2-related genes. Similarly, the CN status for *CDC6*, which is located between *THRA* and *RARA*, was closer related to *TOP2A* than to any other gene tested.

### Lack of concordance between TOP2A FISH status and qPCR CN

**Figure 3:**
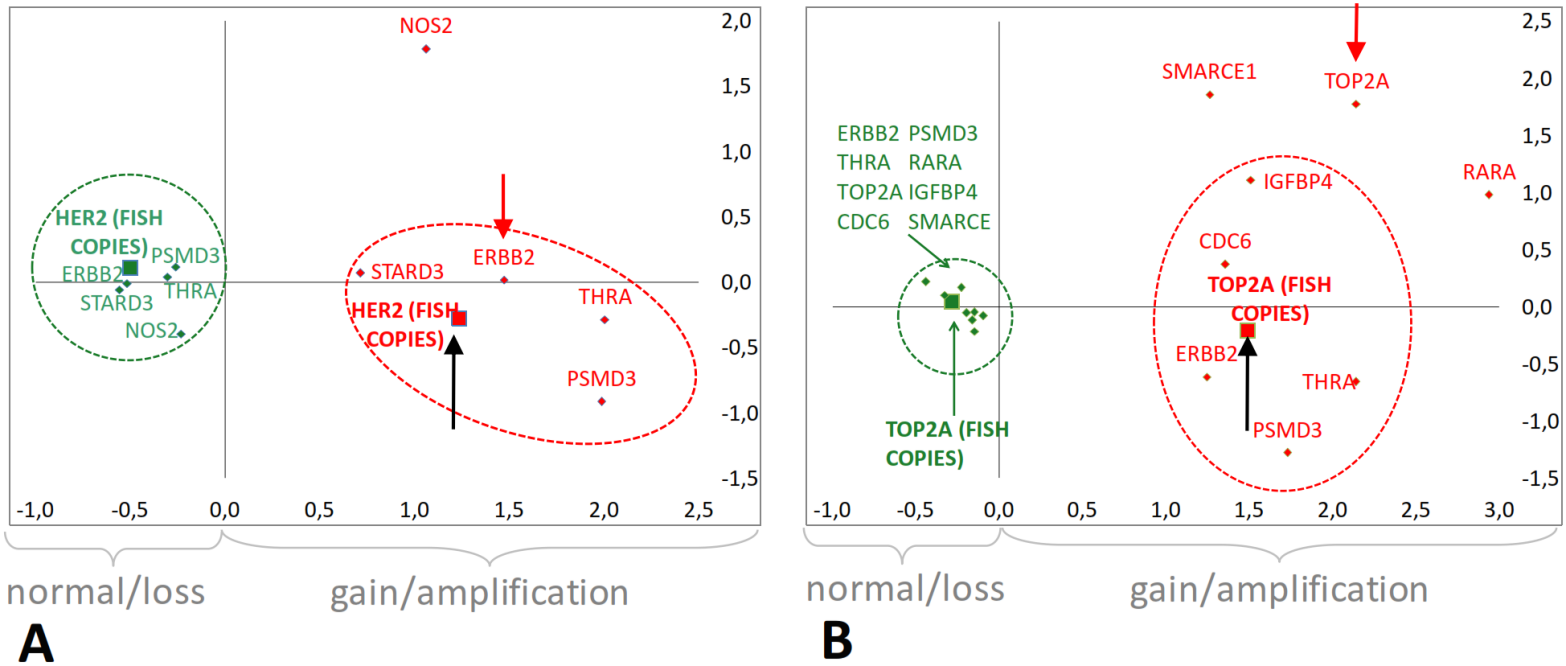
Multiple Correspondence Analyses graphs for comparing gene copies as assessed with qPCR and FISH. **A:** Correspondence of *HER2* FISH (copies) to CN variables (n = 398). **B:** Correspondence of TOP2A FISH (copies) with CN variables co-expression (n = 393). Green: normal or loss; red: gain or amplification.

The lack of *TOP2A* status correlation with its neighboring genes was unexpected. For the evaluation of qPCR CN against HER2 and TOP2A FISH status, individual gene CN status was evaluated in a binary mode as “gain” for >4 maximal copies per gene, which was expected to correspond to gene amplification by FISH assessed as gene/CEN17 ratio or >6 copies. With ratios, FISH HER2 status was concordant with *ERBB2, PSMD3* and *THRA* CN, based on Kappa values; based on both Kappa and McNemar results, concordance was strongest between HER2 FISH and *ERBB2* (**Table 2**). *TOP2A* CN was not concordant with TOP2A FISH, the latter mainly corresponding to *THRA* CN status. Similar results were obtained when assessing qPCR CN status against HER2 and TOP2A FISH copies. With multiple correspondence analysis, HER2 amplified cases with FISH coincided with those exhibiting >4 CN with qPCR for *ERBB2, STARD3, PSMD3* and *THRA*; in comparison, cases called as TOP2A amplified with FISH were not identified as harboring *TOP2A* CN gains with qPCR (**Figure 3**). TOP2A FISH amplified cases had high CN for *ERBB2, PSMD3, THRA, CDC6* and *IGFBP4*.

**Table 2:** Concordance between FISH and qPCR CN classification of chromosome 17 gene status.

		Non-amplified	Amplified	Kappa Value	Kappa 95% CI	P-value (McNemar)*
		HER2 FISH status by ratio				
TP53	no gain	264 (86.8)	91 (91.0)	–0.0500	(–0.132 to 0.031)	<0.001
	gain	40 (13.2)	9 (9.0)			
MAP2K4	no gain	301 (99.7)	96 (96.0)	0.0538	(–.002 to 0.110)	<0.001
	gain	1 (0.3)	4 (4.0)			
NOS2	no gain	302 (83.9)	82 (71.9)	0.1289	(0.032 to 0.226)	0.052
	gain	58 (16.1)	32 (28.1)			
STARD3	no gain	179 (58.9)	6 (6.0)	0.3779	(0.305 to 0.450)	<0.001
	gain	125 (41.1)	94 (94.0)			
ERBB2	no gain	322 (89.4)	22 (19.3)	0.6694	(0.593 to 0.746)	0.052
	gain	38 (10.6)	92 (80.7)			
PSMD3	no gain	295 (96.7)	52 (52.0)	0.5207	(0.420 to 0.621)	<0.001
	gain	10 (3.3)	48 (48.0)			
THRA	no gain	340 (95.0)	67 (58.3)	0.4292	(0.331 to 0.527)	<0.001
	gain	18 (5.0)	48 (41.7)			
CDC6	no gain	267 (87.5)	75 (75.0)	0.1432	(0.039 to 0.247)	0.001
	gain	38 (12.5)	25 (25.0)			
RARA	no gain	349 (97.2)	100 (87.7)	0.1300	(0.047 to 0.213)	<0.001
	gain	10 (2.8)	14 (12.3)			
TOP2A	no gain	343 (96.3)	106 (93.0)	0.0466	(–.023 to 0.116)	<0.001
	gain	13 (3.7)	8 (7.0)			
IGFBP4	no gain	270 (89.1)	85 (85.0)	0.0498	(–.045 to 0.144)	<0.001
	gain	33 (10.9)	15 (15.0)			
SMARCE1	no gain	270 (88.2)	83 (83.8)	0.0524	(–.044 to 0.149)	<0.001
	gain	36 (11.8)	16 (16.2)			
KRT20	no gain	279 (91.5)	90 (91.8)	–0.005	(–.085 to 0.076)	<0.001
	gain	26 (8.5)	8 (8.2)			
STAT3	no gain	268 (88.2)	97 (98.0)	–0.124	(–.178 to –.070)	<0.001
	gain	36 (11.8)	2 (2.0)			
		**TOP2A FISH status by ratio**				
TP53	no gain	295 (88.1)	60 (87.0)	0.0125	(–.086 to 0.110)	0.057
	gain	40 (11.9)	9 (13.0)			
MAP2K4	no gain	330 (99.1)	67 (97.1)	0.0316	(–.033 to 0.096)	<0.001
	gain	3 (0.9)	2 (2.9)			
NOS2	no gain	329 (83.5)	55 (68.8)	0.1405	(0.038 to 0.243)	0.411
	gain	65 (16.5)	25 (31.3)			
STARD3	no gain	172 (51.3)	13 (18.8)	0.1745	(0.108 to 0.241)	<0.001
	gain	163 (48.7)	56 (81.2)			
ERBB2	no gain	318 (80.7)	26 (32.5)	0.3860	(0.292 to 0.480)	<0.001
	gain	76 (19.3)	54 (67.5)			
PSMD3	no gain	304 (90.5)	43 (62.3)	0.3006	(0.180 to 0.421)	0.248
	gain	32 (9.5)	26 (37.7)			
THRA	no gain	368 (93.6)	39 (48.8)	0.4825	(0.374 to 0.591)	0.103
	gain	25 (6.4)	41 (51.3)			
CDC6	no gain	304 (90.5)	38 (55.1)	0.3667	(0.248 to 0.486)	0.550
	gain	32 (9.5)	31 (44.9)			
RARA	no gain	386 (98.2)	63 (78.8)	0.2699	(0.156 to 0.384)	<0.001
	gain	7 (1.8)	17 (21.3)			
TOP2A	no gain	378 (96.9)	71 (88.8)	0.1156	(0.018 to 0.214)	<0.001
	gain	12 (3.1)	9 (11.3)			
IGFBP4	no gain	306 (91.6)	49 (71.0)	0.2343	(0.113 to 0.355)	0.022
	gain	28 (8.4)	20 (29.0)			
SMARCE1	no gain	304 (90.5)	49 (71.0)	0.2157	(0.096 to 0.335)	0.075
	gain	32 (9.5)	20 (29.0)			
KRT20	no gain	309 (92.5)	60 (87.0)	0.0696	(–.035 to 0.174)	<0.001
	gain	25 (7.5)	9 (13.0)			
STAT3	no gain	305 (91.3)	60 (87.0)	0.0531	(–.050 to 0.156)	0.001
	gain	29 (8.7)	9 (13.0)			

Non-amplified: HER2/CEP17 ratio <2.2, TOP2A/CEP17 ratio <2;

*McNemar higher P-values indicate higher agreement.

### Patterns of chr17 gene CN

**Figure 4:**
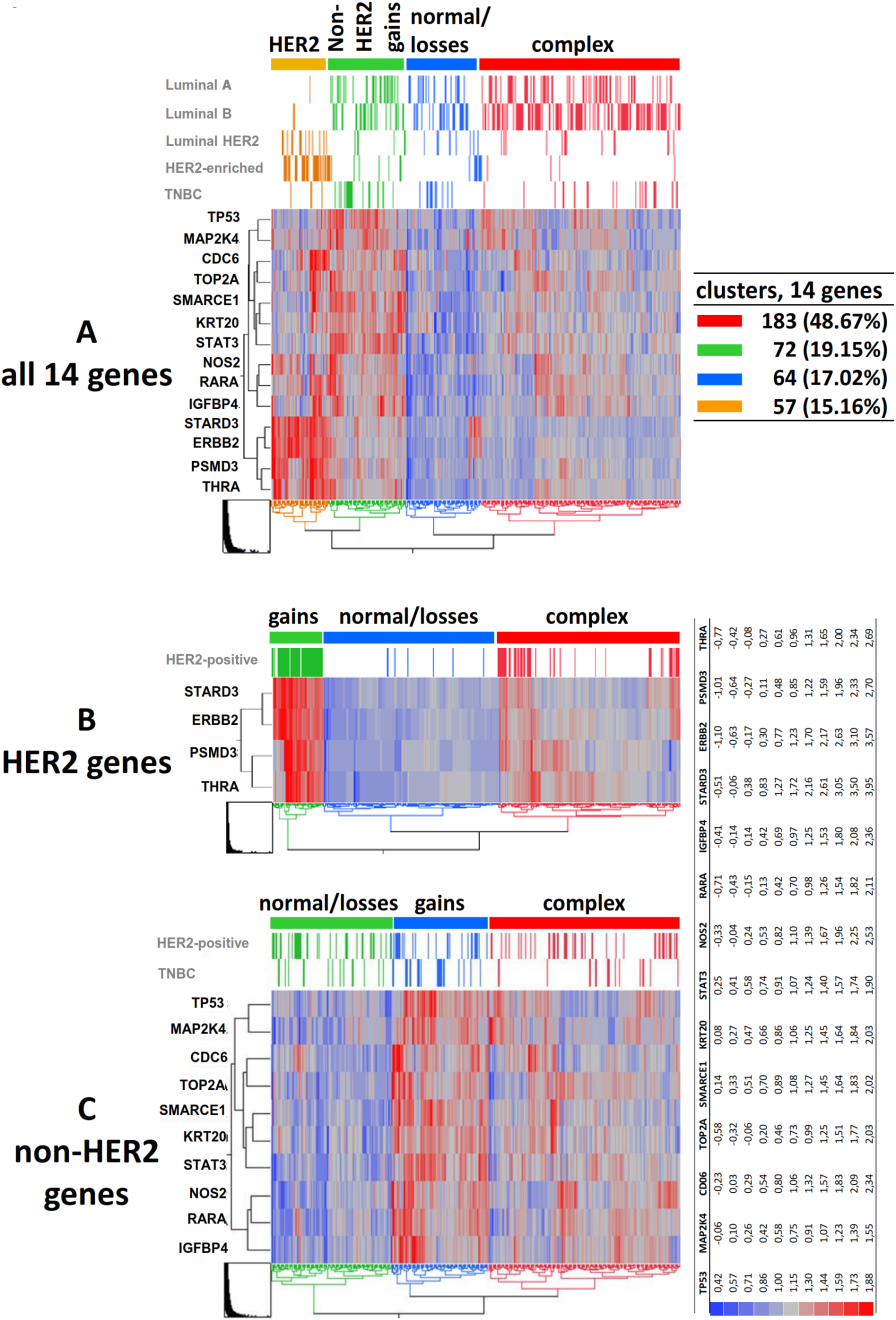
Hierarchical clustering of log transformed qPCR copy number (CN) values for chr17 genes in association with relevant breast cancer subtypes. **A:** clustering of all 14 genes was informative in 376 cases and revealed 4 main CN patterns, out of which high CN for the HER2-related genes form the HER2 cluster and are predominantly found in HER2-positive (Luminal-HER2 and HER2-enriched) tumors; gains for the HER2 gene itself (ERBB2) mostly occur in parallel with STARD3. Luminal A and B, as well as TNBC are evenly represented in the non-HER2 clusters. **B:** If analyzing CN for the 4 HER2-related genes only, 36 of 93 tumors (38.7%) have non-continuous gains for these genes (complex pattern, red cluster) while the rest exhibit continuous gains in at least two genes, predominantly STARD3 and ERBB2 (gains, green cluster). **C:** Clustering of the non-HER2-related gene CN, i.e., 10 of 14 genes excluding STARD3, ERBB2, PSMD3 and THRA, yields 3 main clusters with equal representation of all subtypes (distribution of HER2-positive tumors and TNBC is shown). Cluster legend: natural log 0.69 corresponds to 2 copies; log 1.38 to 4 copies.

Since chr17 gene CN aberrations did not occur singly but were related to each other, we next examined CN patterns. Hierarchical clustering of log transformed CN for the 14 genes (**Figure 4A**) revealed four main clusters based on the distance dendrogram. These clusters distinguished tumors with HER2-related gains, non-HER2-related gains, low CN (normal or losses), and complex changes. The cluster with HER2-related gains almost exclusively included HER2-positive tumors (luminal-HER2 and HER2-enriched) prompting for separate analysis of HER2-related and non-HER2 CN patterns in order to avoid HER2 bias. HER2-related gene CN clustering revealed *STARD3, ERBB2, PSMD3* and *THRA* CN gains, complex changes and normal status (including possible losses) in three distinct groups (**Figure 4B**). Similarly, clustering of the remaining 10 non-HER2-related gene CN, again yielded three main patterns with gains, complex changes, and, normal/losses (**Figure 4C**).

HER2-related gene CN clusters showed the expected strong associations with menopausal and tumor hormonal status, histological grade, tumor HER2 and TOP2A FISH status, and, with HER2-positive subtypes (**Table 3**). Non-HER2-related complex CN changes were significantly more often in larger (39/109, 52.3%) as compared to smaller tumors (138/264, 35.8%), while gains were less often in hormone receptor positive (52/284, 18.3%) as compared to negative tumors (34/89, 38.2%). Non-HER2-related clusters showed a similar distribution in luminal tumors, which differed from that observed in HER2-enriched and triple-negative breast cancer (TNBC).

**Table 3:** HER2- and non-HER2-related gene CN clusters comparisons with standard clinicopathological parameters.

	Non-HER2 gene CN clusters				HER2-related gene CN clusters			
	Gains	Complex	Normal/Losses	P-value	HER2amp	Complex	Normal/Losses	P-value
Total cluster N = 376	92 (24.47)	178 (47.34)	106 (28.19)		55 (14.63)	166 (44.15)	153 (40.69)	
**Age**								
34–50	26 (30.2)	63 (35.6)	34 (30.9)	0.592	16 (32.0)	54 (30.7)	66 (38.4)	0.106
<34	2 (2.3)	5 (2.8)	6 (5.5)			4 (2.3)	9 (5.2)	
>50	58 (67.4)	109 (61.6)	70 (63.6)		34 (68.0)	118 (67.0)	97 (56.4)	
**Menopausal Status**								
Post	60 (69.8)	103 (58.2)	64 (58.2)	0.155	33 (66.0)	117 (66.5)	88 (51.2)	0.009
Pre	26 (30.2)	74 (41.8)	46 (41.8)		17 (34.0)	59 (33.5)	84 (48.8)	
**Type of surgery**								
BCS (conserving)	34 (39.5)	60 (33.9)	42 (38.2)	0.609	16 (32.0)	63 (35.8)	68 (39.5)	0.571
MRM (modified radical)	52 (60.5)	117 (66.1)	68 (61.8)		34 (68.0)	113 (64.2)	104 (60.5)	
**Tumor size**								
≤2 cm	29 (33.7)	39 (22.0)	41 (37.3)	0.013	13 (26.0)	49 (27.8)	54 (31.4)	0.668
>2 cm	57 (66.3)	138 (78.0)	69 (62.7)		37 (74.0)	127 (72.2)	118 (68.6)	
**Number of positive nodes**								
1–3	43 (50.0)	80 (45.2)	47 (42.7)	0.592	23 (46.0)	85 (48.3)	76 (44.2)	0.744
>3	43 (50.0)	97 (54.8)	63 (57.3)		27 (54.0)	91 (51.7)	96 (55.8)	
**Histological grade**								
I–II	39 (45.3)	75 (42.4)	55 (50.0)	0.451	14 (28.0)	74 (42.0)	93 (54.1)	0.002
III-Undifferentiated	47 (54.7)	102 (57.6)	55 (50.0)		36 (72.0)	102 (58.0)	79 (45.9)	
**Histological type**								
Comedo	1 (1.2)	7 (4.0)	2 (1.8)	0.473	2 (4.0)	2 (1.1)	7 (4.1)	0.180
Inflammatory		1 (0.6)	2 (1.8)			2 (1.1)	1 (0.6)	
Invasive ductal	73 (84.9)	143 (80.8)	85 (77.3)		46 (92.0)	147 (83.5)	127 (73.8)	
Invasive lobular	7 (8.1)	16 (9.0)	9 (8.2)		2 (4.0)	14 (8.0)	18 (10.5)	
Medullary	2 (2.3)	1 (0.6)	4 (3.6)			2 (1.1)	6 (3.5)	
Mixed	3 (3.5)	9 (5.1)	8 (7.3)			9 (5.1)	12 (7.0)	
Papillary							1 (0.6)	
**ER/PgR status**								
Negative	34 (39.5)	29 (16.4)	26 (23.6)	<0.001	32 (64.0)	44 (25.0)	20 (11.6)	<0.001
Positive	52 (60.5)	148 (83.6)	84 (76.4)		18 (36.0)	132 (75.0)	152 (88.4)	
**HER2 status**								
Negative	62 (72.1)	135 (77.1)	79 (72.5)	0.565	5 (10.0)	128 (73.6)	162 (94.7)	<0.001
Positive (3+ or amplified)	24 (27.9)	40 (22.9)	30 (27.5)		45 (90.0)	46 (26.4)	9 (5.3)	
**Ki67**								
High (≥14%)	52 (61.2)	121 (68.4)	79 (72.5)	0.243	35 (71.4)	127 (72.2)	105 (61.4)	0.083
Low (<14%)	33 (38.8)	56 (31.6)	30 (27.5)		14 (28.6)	49 (27.8)	66 (38.6)	
**TOP2A (FISH)**								
Amplified	16 (18.6)	17 (9.6)	10 (9.1)	0.128	24 (48.0)	17 (9.7)	5 (2.9)	0.000
Deleted	5 (5.8)	6 (3.4)	8 (7.3)		7 (14.0)	5 (2.8)	8 (4.7)	
Equivocal	2 (2.3)	1 (0.6)	1 (0.9)			3 (1.7)	1 (0.6)	
Non-amplified	63 (73.3)	153 (86.4)	91 (82.7)		19 (38.0)	151 (85.8)	158 (91.9)	
**TOP2A (FISH) binary**								
Amplified	16 (18.6)	17 (9.6)	10 (9.1)	0.064	24 (48.0)	17 (9.7)	5 (2.9)	<0.001
Non-amplified	70 (81.4)	160 (90.4)	100 (90.9)		26 (52.0)	159 (90.3)	167 (97.1)	
**Subtypes (IHC4)**								
Luminal A	24 (27.9)	46 (26.3)	24 (22.0)	0.006	1 (2.0)	40 (23.0)	61 (35.7)	<0.001
Luminal B	21 (24.4)	76 (43.4)	42 (38.5)		1 (2.0)	64 (36.8)	81 (47.4)	
Luminal-HER2	7 (8.1)	24 (13.7)	17 (15.6)		16 (32.0)	26 (14.9)	9 (5.3)	
HER2-enriched	17 (19.8)	16 (9.1)	13 (11.9)		29 (58.0)	20 (11.5)		
TNBC	17 (19.8)	13 (7.4)	13 (11.9)		3 (6.0)	24 (13.8)	20 (11.7)	
**Adjuvant RT**								
No	23 (27.1)	37 (22.3)	24 (22.2)	0.659	11 (23.4)	42 (24.4)	37 (22.4)	0.911
Yes	62 (72.9)	129 (77.7)	84 (77.8)		36 (76.6)	130 (75.6)	128 (77.6)	
**Adjuvant HT**								
No	22 (25.6)	31 (18.2)	27 (25.2)	0.260	20 (40.0)	43 (24.9)	27 (16.4)	0.002
Yes	64 (74.4)	139 (81.8)	80 (74.8)		30 (60.0)	130 (75.1)	138 (83.6)	

### Diverse effects of the same gene CN patterns on patient outcome according to tumor subtype, nodal status and the applied type of surgery

DFS and OS did not differ significantly between treatment groups. At a median follow-up of 101.7 months (range: 0.1–161.4), the 5-year DFS rates were 77.1% and 69.3%, while the OS rates were 88.9% and 83.2%, for the E-T-CMF and E-CMF arms, respectively. Clinicopathological parameters significantly implicated in patient outcome, as revealed with log-rank testing for OS, were type of surgery (favorable for BCS, p = 0.0498), nodal status (favorable for 1–3 positive nodes, p = 0.0003), and IHC4 subtypes (worst for TNBC, p = 0.0098); type of surgery (p = 0.0389) and nodal status (p<0.0001) for DFS. Single gene CN status (5 scale) was not associated with patient outcome, neither were CN clusters with all 14 genes or with the 10 non-HER2-related genes. Tumors with *STARD3, ERBB2, PSMD3* and *THRA* CN gains were associated with longer OS, with 94.0% of the patients at risk at 5 years as compared to 89.8% and 85.1% for tumors with mixed or normal patterns of the same genes (p = 0.0354). HER2-related CN clusters were not associated with time to relapse.

**Figure 5:**
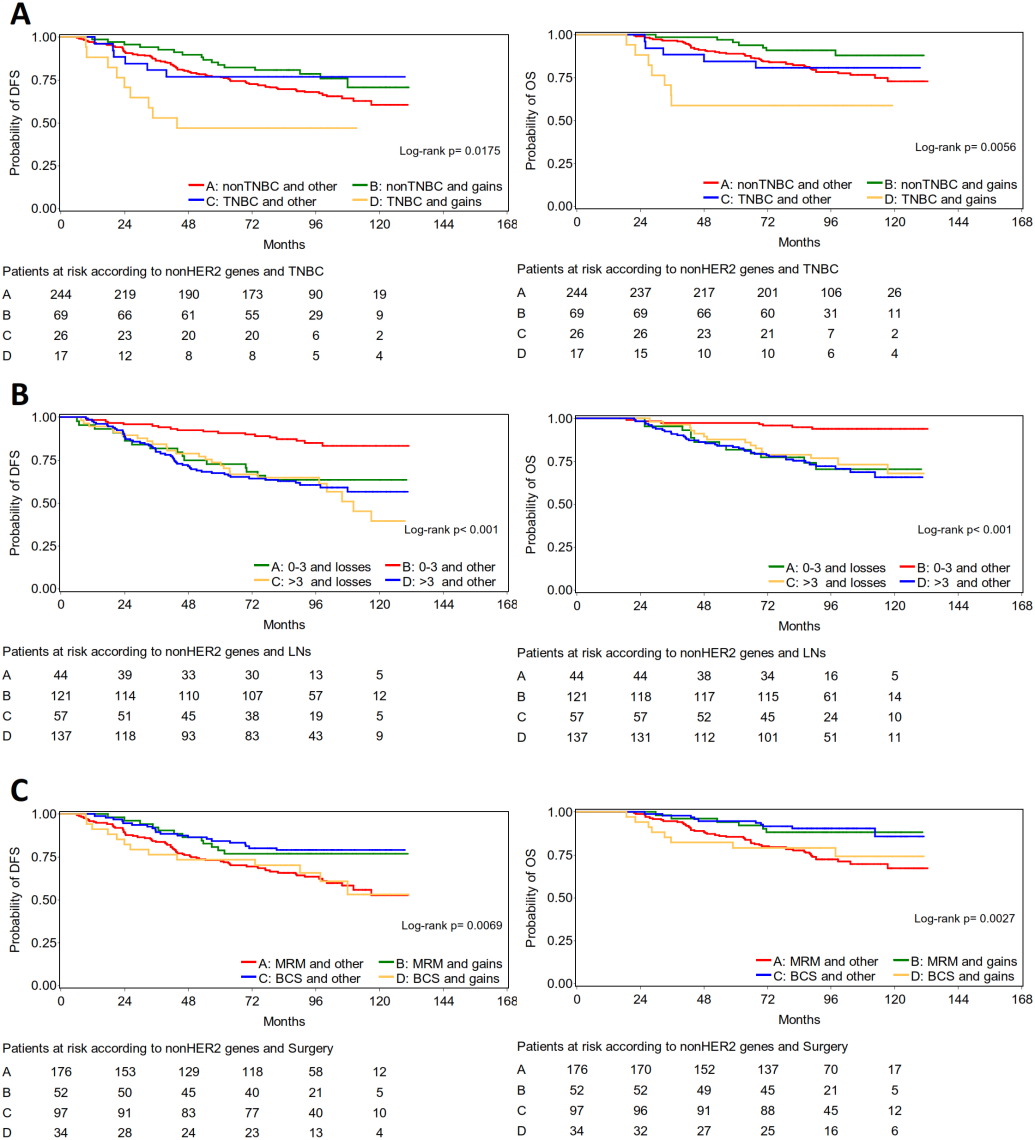
Significant interactions between non-HER2 gene CN clusters and clinicopathologic parameters affecting patient survival. In **A,** among patients with non-TNBC tumors those with non-HER2 gene CN gains fare best; among patients with TNBC, those with non-HER2 gains fare worse. In **B,** patients with favorable nodal status (1–3 metastatic lymph nodes) and tumors with non-HER2-gene CN losses have similarly bad outcome as those with unfavorable nodal status. In **C,** non-HER2-gene CN gains strikingly confer the opposite outcome to patients who have undergone modified radical mastectomy (MRM) as compared to breast-conserving surgery (BCS).

Chr17 gene CN patterns for all 14 genes, HER2-related, and non-HER2-related clusters were significantly implicated in patient outcome when examined in combination with clinicopathological parameters at univariate analysis (**Tables S4 and S5**). In particular, non-HER2-related CN gains conferred significantly increased risk for relapse and death in patients with TNBC (DFS: Hazard Ratio [HR] 3.5, 95% confidence interval [CI] 1.5–7.9; OS, HR 6.0, 95% CI 2.1–17.1) but not with other subtypes, while no differences were observed in the outcome of patients with tumors exhibiting complex or normal non-HER2-related CN patterns, regardless of the underlying subtype (interaction Wald’s p = 0.0082 for DFS; p = 0.0092 for OS) (**Figure 5A**). Among patients in the favorable prognosis group with 1–3 positive nodes, the outcome of those with non-HER2-related CN losses was in fact unfavorable (DFS, HR 2.9, 95% CI 1.5–5.6; OS, HR 5.4, 95% CI 2.1–13.8), similar to the outcome of patients with >3 positive nodes and tumors with any other non-HER2-related CN pattern (interaction p = 0.0172 for DFS; p = 0.0010 for OS) (**Figure 5B**). Finally, non-HER2-related CN gains in tumors from patients who underwent BCS conferred increased risk for relapse and death (DFS HR 2.1, 95% CI 1.1–4.3; OS HR 2.6, 95% CI 1.0–6.6) but a favorable outcome to those who underwent MRM (DFS HR 0.5, 95% CI 0.3–1.0; OS HR 0.4, 95% CI 0.2–0.9). BCS was associated with favorable outcome as compared to MRM only in the absence of non-HER2-related CN gains (interaction p = 0.0039 for DFS; p = 0.0036 for OS) (**Figure 5C**).

**Figure 6:**
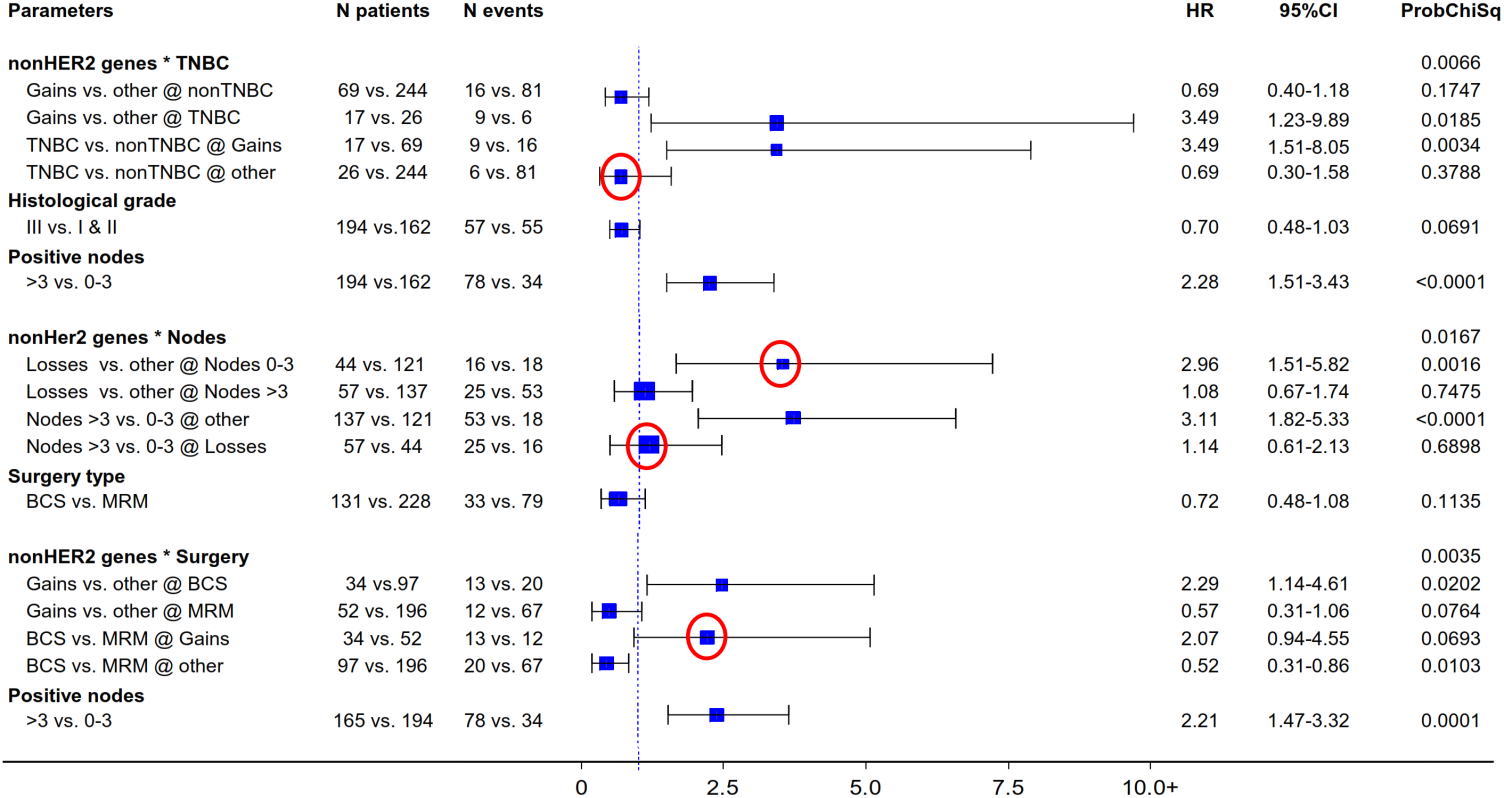
Multivariate analysis models involving the main significant non-HER2-gene CN genotype effects on patient DFS. Red circles: novel findings with potentially significant clinical implications, worthy pursuing for further validation. CN gains in non-HER2 genes on chr17 confer inverse risk for relapse in patients with TNBC and non-TNBC tumors, and in those who have undergone BCS in comparison to MRM. Similarly, the favorable 1–3 nodal status confers the same risk for relapse as the unfavorable disease with >3 positive nodes, if tumors have non-HER2-gene CN losses.

**Figure 7:**
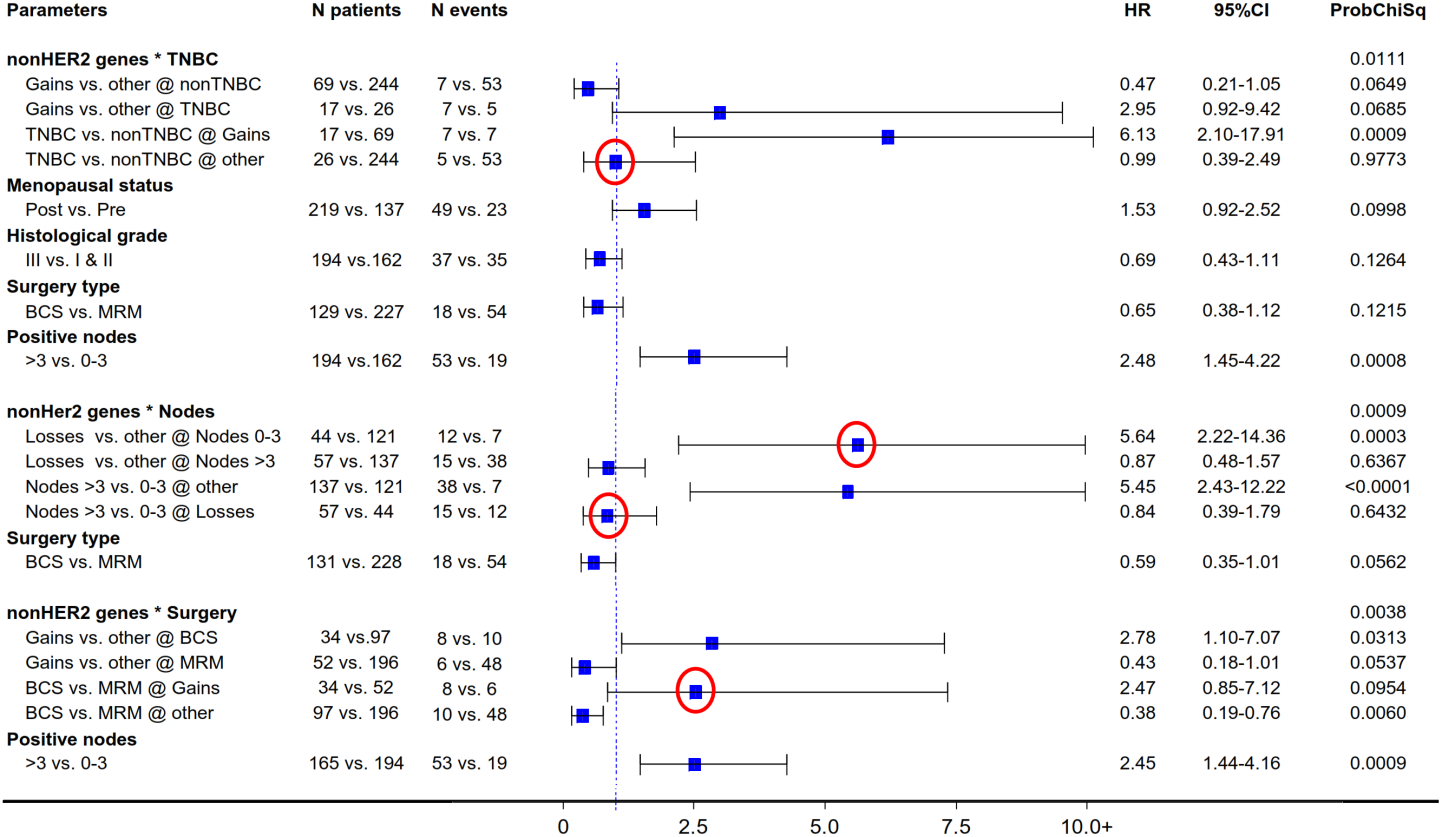
Multivariate analysis models involving the main significant non-HER2-gene CN pattern effects on patient OS. Findings as for DFS in Figure 6.

The described non-HER2-related CN cluster interactions remained significant upon multivariate analysis for both DFS (**Figure 6**) and OS (**Figure 7**). As shown, non-TNBC and TNBC lacking CN gains within this group of 10 genes shared similar outcomes, while the unfavorable behavior of TNBC was independently predicted by the presence of CN gains of the same genes. Low non-HER2-gene CN independently predicted for an unfavorable outcome among patients with 1–3 metastatic nodes, which did not differ from that of patients with >3 metastatic nodes. And, the presence of non-HER2-related CN gains with respect to BCS and MRM independently predicted for an unfavorable and favorable outcome, respectively.

## Discussion

In accordance to previous reports involving assessment of multiple chr17 genes or regions, this study once more portrays the complexity of chr17 alterations in breast cancer. There was no single tumor with CN gains for all 14 genes, further supporting the rarity of chr17 polysomy, i.e., increased copies of the entire chromosome, as also shown with array-based comparative genomic hybridization (aCGH) [4], [6], [7], alternative FISH approaches [11] and MLPA [25]. In order to compare the present data on gene dosage with relevant literature findings, however, we need to consider technical characteristics of the methods applied for this purpose. qPCR targets very short amplicons (up to 120 bp) within individual genes. In comparison, the BAC probes used for FISH and aCGH span hundreds of kilobases. Therefore, results by FISH and aCGH for chr17 may be highly concordant [26], but qPCR CN merely delineate those by aCGH [27]. Further, differences in the number, selection and application of reference genes for predicting CN, type of probes used, as well as differences in the selection of the amplicons targeted with qPCR may also yield different results with various qPCR approaches. The above explain, for example, why *HER2* FISH and qPCR *ERBB2* CN status were only fairly concordant in the present study, as compared to the strong concordance reported with a different qPCR approach [28].

In line with the only study so far, using qPCR for assessing the status of multiple genes on 17q [8], CN of the genes surrounding *ERBB2*, i.e., *STARD3, PSMD3* and *THRA* mostly increased in parallel. *STARD3* and *ERBB2* exhibited the highest CN values observed in this study, mostly but not always coinciding. Thus, *STARD3* may rather be at the edge of the smallest region of amplification described for the HER2 amplicon [29] and not within this region as stated by others [8], [30]–[32]. *STARD3*, a gene involved in steroidogenesis [33], is overexpressed in HER2-positive cancer cells [34] and it seems worthy investigating whether it contributes to the lipid-rich phenotype described for HER2-positive tumors [35]. *THRA*, another metabolic gene experimentally shown to interfere with insulin resistance [36], was also frequently co-amplified along with *ERBB2* and *STARD3*. Hence, *STARD3* and *THRA* may represent additional targets for therapeutic interventions in HER2-positive cancers.

Unexpectedly, qPCR *TOP2A* CN were not associated with *TOP2A* FISH, *HER2* FISH and *ERBB2* CN status, which may partially explain the discrepancy between FISH *TOP2A* amplification and respective mRNA and protein expression described partially for this series [37] and elsewhere [38]. Instead, *TOP2A* CN did associate with *THRA* and *CDC6* CN. Concordance with TOP2A FISH has been described for qPCR with hybridization probes [39] but respective reports with hydrolysis (Taqman) probes are particularly missing. With classic FISH, *TOP2A* amplification is rare in *HER2* non-amplified tumors, which was noticed for the present series, as well. However, *TOP2A* gene gains with qPCR have been independently reported as unrelated to *HER2* FISH status [40], [41], in line with the present findings. This discrepancy may be explained by (a) the presence of breakpoints and possible rearrangements within the 17q21 region covered by the FISH probe targeting *RARA* and *IGFBP4* in addition to *TOP2A*; (b) the break-fusion-bridge pattern, proposed for the development of *TOP2A* amplification, has been suggested by using probes covering the *THRA* region, as well [42]; (c) the use of ratios instead of copies with FISH; and, (d) the different specificity/sensitivity of the *TOP2A* FISH assays [43]. Overall, due to the low number of *TOP2A* copies observed with both FISH [44] and qPCR, the use of gene ratios vs. the centromeric probe should probably be replaced by gene copies when evaluating amplification, as was recently also suggested for the evaluation of the *HER2* gene with FISH [45].

Considering that the CN clusters reflect different levels and types of chr17 instability, this study supports the notion that HER2-positive tumors exhibit different patterns of CIN [46]. Herein we found that compact HER2 amplicon gains are more common in HER2-enriched, while complex gains are the main pattern in luminal-HER2 tumors. Interestingly, HER2-positive patients with compact HER2 amplicon gains had similar time-to-relapse, but significantly prolonged survival as compared to patients with normal or complex HER2 amplicon gene CN. Since all HER2-positive patients had received trastuzumab upon relapse, the observed favorable survival for those with the compact gains pattern might be attributed to this drug, as seen in patients with high level *HER2* gene amplification [47] and HER2-enriched tumors [48].

The different CN patterns of the 10 genes outside the core HER2 amplicon may reflect the presence of various degrees and patterns of CIN among breast cancer subtypes, as observed with different methods [6], [7], [49]–[51]. The distribution of these patterns differed in luminal and non-luminal subtypes, indicating an estrogen-related background playing a role in the development of these complex patterns, and supporting previous evidence that the luminal B subtype is the most unstable one among ER-positive tumors [49]. Importantly, the obtained chr17 CN patterns predicted for good prognosis in patients with standard unfavorable characteristics and *vice versa*. Thus, the favorable prognosis in patients with 1 to 3 metastatic lymph nodes was observed only in those with gains and complex CN patterns of non-HER2 genes. By contrast, prognosis for patients with the same favorable nodal status, but with tumors exhibiting predominantly losses of non-HER2 genes, was similar to patients with unfavorable nodal status. This seems to contradict a previous report on unstable tumors with negative nodal status faring worse [51]. However, the present chr17 patterns are not comparable to the genomic index in the latter study, while in the present series, there were no patients with negative nodal status. Hence, the two studies actually show that different patterns of instability have diverse effects on the outcome of patients with the same nodal status.

The impact of the same chromosomal stability status, as determined by different methods [49], [50], [52], was inversely related to the outcome of patients with ER-positive as compared to ER-negative tumors, the behavior of which is driven by different pathways [53]. In a similar manner, among the 5 subtypes considered in the present study, TNBC patients had overall the worst outcome, which concerned though tumors with the chr17 non-HER2 CN gain pattern only. In fact, TNBC patients with losses or complex non-HER2 CN patterns had a relatively more favorable outcome, similar to non-TNBC, mostly ER-positive patients with chr17 non-HER2 gains in their tumors. To our knowledge this is a novel finding that would be worth validating in larger patient series; distinguishing between favorable and unfavorable TNBC, based on chr17 or genome wide CIN patterns, might aid in modifying treatment selection accordingly.

In addition, with respect to the applied surgery type, BCS was overall associated with better survival, which was recently presented as an effect of nodal status on long term follow-up [54]. Here we show that the outcome of patients who underwent BCS or MRM may independently be predicted by the chr17 CN pattern in the tumor. Patients with tumors exhibiting chr17 non-HER2 losses or complex patterns and patients with chr17 gains shared the same favorable outcome on BCS and MRM. By contrast, the same unfavorable outcome was observed for patients who received BCS for tumors with chr17 gains and MRM for tumors with chr17 losses or complex patterns. This is also a novel finding with potential clinical relevance, which, if validated, would prompt for core needle biopsy before surgery. Relevant data exist for biomarker testing in the neoadjuvant setting [55]. It remains questionable though, whether this type of biopsy would be appropriate for small sized tumors, as was the case for about 1/3 of the patients in the present series.

Overall, the herein presented chr17 CN patterns may reflect the presence of different CIN types that probably evolve on the ground of different DNA repair defects. Thus, next to the extent of CIN [1], the type of these changes also seems to affect disease course. Whether chr17 instability is a surrogate for the condition in the whole genome, as previously stated [10], and whether it interferes with anthracyclines or taxanes for patient outcome [1], [56] cannot be answered by the present study. It is important to note though, that chr17 CN alterations outside the core HER2 amplicon occur in HER2-negative tumors and interfere with the outcome of breast cancer patients with established prognostic disease characteristics. Re-interpretation of traditionally obtained chr17 gene results with FISH, classification and typing of CIN with appropriately standardized methods and understanding its diverse effects on disease behavior seem mandatory for efficiently assessing patients with high-risk breast cancer.

## Supporting Information

Figure S1 **qPCR CN 5-scale classification.** The number of informative samples and copy number (CN) distribution per category is shown. Genes are in lanes, classification for 5′ and 3′ amplicons, and for maximal CN is in rows. Red circles: significant discrepant classification between 5′ and 3′ ends.
(DOC)

Figure S2 **CN value distribution upon logarithmic transformation (natural logarithm).** **a,** 5′ end; **b,** 3′ end; **c,** highest value of the two. CN ranged between 2–4 in the majority of tumors, indicating low gains and/or DNA replication in the majority of cells.
(DOC)

Figure S3 **Clustered correlations for 5′ and 3′ end copy number (CN) values and for maximal CN.** **A,** log transformed; **B,** 5-scale classification. **C,** maximal CN (5-scale) Similar to log transformed CN in Figure 1, core HER2 amplicon genes (STARD3, ERBB2, PSMD3, THRA) cluster together for both amplicons per gene and for maximal CN. CN for NOS2, RARA and IGFBP4 also correlate to each other, while TOP2A status is only vaguely related to these genes.
(DOC)

Table S1 **qPCR assays for the assessment of chromosome 17 gene CN.**
(XLS)

Table S2 **Description of predicted CN for each assay per gene and of the finally used values (Maximal CN [Max CN]).**
(XLS)

Table S3 **Max CN associations with clinicopathological parameters.**
(XLS)

Table S4 **Impact on patient disease-free survival (DFS) of interactions between chromosome 17 gene CN clusters and clinicopatholofical parameters (Univariate Cox).**
(XLS)

Table S5 **Impact on patient overall survival (OS) of interactions between chromosome 17 gene CN clusters and clinicopatholofical parameters (Univariate Cox).**
(XLS)

## References

[1] McGranahan N, Burrell RA, Endesfelder D, Novelli MR, Swanton C (2012) Cancer chromosomal instability: therapeutic and diagnostic challenges. EMBO Rep 13: 528–538. 22595889 10.1038/embor.2012.61 PMC3367245

[2] Hanahan D, Weinberg RA (2011) Hallmarks of cancer: the next generation. Cell 144: 646–674. 21376230 10.1016/j.cell.2011.02.013

[3] Wolff AC, Hammond ME, Hicks DG, Dowsett M, McShane LM, et al. (2013) Recommendations for Human Epidermal Growth Factor Receptor 2 Testing in Breast Cancer: American Society of Clinical Oncology/College of American Pathologists Clinical Practice Guideline Update. Arch Pathol Lab Med. 10.5858/arpa.2013-0953-SA PMC4086638 24099077

[4] Arriola E, Marchio C, Tan DS, Drury SC, Lambros MB, et al (2008) Genomic analysis of the HER2/TOP2A amplicon in breast cancer and breast cancer cell lines. Lab Invest 88: 491–503. 18332872 10.1038/labinvest.2008.19

[5] Kytola S, Rummukainen J, Nordgren A, Karhu R, Farnebo F, et al (2000) Chromosomal alterations in 15 breast cancer cell lines by comparative genomic hybridization and spectral karyotyping. Genes Chromosomes Cancer 28: 308–317. 10862037 10.1002/1098-2264(200007)28:3<308::aid-gcc9>3.0.co;2-b

[6] Marchio C, Lambros MB, Gugliotta P, Di Cantogno LV, Botta C, et al (2009) Does chromosome 17 centromere copy number predict polysomy in breast cancer? A fluorescence in situ hybridization and microarray-based CGH analysis. J Pathol 219: 16–24. 19670217 10.1002/path.2574

[7] Yeh IT, Martin MA, Robetorye RS, Bolla AR, McCaskill C, et al (2009) Clinical validation of an array CGH test for HER2 status in breast cancer reveals that polysomy 17 is a rare event. Mod Pathol 22: 1169–1175. 19448591 10.1038/modpathol.2009.78

[8] Lamy PJ, Fina F, Bascoul-Mollevi C, Laberenne AC, Martin PM, et al (2011) Quantification and clinical relevance of gene amplification at chromosome 17q12-q21 in human epidermal growth factor receptor 2-amplified breast cancers. Breast Cancer Res 13: R15. 21288332 10.1186/bcr2824 PMC3109584

[9] Kallioniemi OP, Kallioniemi A, Kurisu W, Thor A, Chen LC, et al (1992) ERBB2 amplification in breast cancer analyzed by fluorescence in situ hybridization. Proc Natl Acad Sci U S A 89: 5321–5325. 1351679 10.1073/pnas.89.12.5321 PMC49283

[10] Munro AF, Twelves C, Thomas JS, Cameron DA, Bartlett JM (2012) Chromosome instability and benefit from adjuvant anthracyclines in breast cancer. Br J Cancer 107: 71–74. 22644297 10.1038/bjc.2012.232 PMC3389422

[11] Tse CH, Hwang HC, Goldstein LC, Kandalaft PL, Wiley JC, et al (2011) Determining true HER2 gene status in breast cancers with polysomy by using alternative chromosome 17 reference genes: implications for anti-HER2 targeted therapy. J Clin Oncol 29: 4168–4174. 21947821 10.1200/JCO.2011.36.0107

[12] Fountzilas G, Skarlos D, Dafni U, Gogas H, Briasoulis E, et al (2005) Postoperative dose-dense sequential chemotherapy with epirubicin, followed by CMF with or without paclitaxel, in patients with high-risk operable breast cancer: a randomized phase III study conducted by the Hellenic Cooperative Oncology Group. Ann Oncol 16: 1762–1771. 16148021 10.1093/annonc/mdi366

[13] Fountzilas G, Dafni U, Gogas H, Linardou H, Kalofonos HP, et al (2008) Postoperative dose-dense sequential chemotherapy with epirubicin, paclitaxel and CMF in patients with high-risk breast cancer: safety analysis of the Hellenic Cooperative Oncology Group randomized phase III trial HE 10/00. Ann Oncol 19: 853–860. 18042835 10.1093/annonc/mdm539

[14] Gogas H, Dafni U, Karina M, Papadimitriou C, Batistatou A, et al (2012) Postoperative dose-dense sequential versus concomitant administration of epirubicin and paclitaxel in patients with node-positive breast cancer: 5-year results of the Hellenic Cooperative Oncology Group HE 10/00 phase III Trial. Breast Cancer Res Treat 132: 609–619. 22187126 10.1007/s10549-011-1913-4

[15] Fountzilas G, Dafni U, Bobos M, Batistatou A, Kotoula V, et al (2012) Differential Response of Immunohistochemically Defined Breast Cancer Subtypes to Anthracycline-Based Adjuvant Chemotherapy with or without Paclitaxel. PLoS One 7: e37946. 22679488 10.1371/journal.pone.0037946 PMC3367950

[16] Fountzilas G, Dafni U, Bobos M, Kotoula V, Batistatou A, et al (2013) Evaluation of the prognostic role of centromere 17 gain and HER2/topoisomerase II alpha gene status and protein expression in patients with breast cancer treated with anthracycline-containing adjuvant chemotherapy: pooled analysis of two Hellenic Cooperative Oncology Group (HeCOG) phase III trials. BMC Cancer 13: 163. 23537287 10.1186/1471-2407-13-163 PMC3621498

[17] Wolff AC, Hammond ME, Schwartz JN, Hagerty KL, Allred DC, et al (2007) American Society of Clinical Oncology/College of American Pathologists guideline recommendations for human epidermal growth factor receptor 2 testing in breast cancer. J Clin Oncol 25: 118–145. 17159189 10.1200/JCO.2006.09.2775

[18] Hammond ME, Hayes DF, Dowsett M, Allred DC, Hagerty KL, et al (2010) American Society of Clinical Oncology/College Of American Pathologists guideline recommendations for immunohistochemical testing of estrogen and progesterone receptors in breast cancer. J Clin Oncol 28: 2784–2795. 20404251 10.1200/JCO.2009.25.6529 PMC2881855

[19] Cheang MC, Chia SK, Voduc D, Gao D, Leung S, et al (2009) Ki67 index, HER2 status, and prognosis of patients with luminal B breast cancer. J Natl Cancer Inst 101: 736–750. 19436038 10.1093/jnci/djp082 PMC2684553

[20] Press MF, Sauter G, Buyse M, Bernstein L, Guzman R, et al (2011) Alteration of topoisomerase II-alpha gene in human breast cancer: association with responsiveness to anthracycline-based chemotherapy. J Clin Oncol 29: 859–867. 21189395 10.1200/JCO.2009.27.5644 PMC3068060

[21] Vanden Bempt I, Van Loo P, Drijkoningen M, Neven P, Smeets A, et al (2008) Polysomy 17 in breast cancer: clinicopathologic significance and impact on HER-2 testing. J Clin Oncol 26: 4869–4874. 18794552 10.1200/JCO.2007.13.4296

[22] Knoop AS, Knudsen H, Balslev E, Rasmussen BB, Overgaard J, et al (2005) retrospective analysis of topoisomerase IIa amplifications and deletions as predictive markers in primary breast cancer patients randomly assigned to cyclophosphamide, methotrexate, and fluorouracil or cyclophosphamide, epirubicin, and fluorouracil: Danish Breast Cancer Cooperative Group. J Clin Oncol 23: 7483–7490. 16234514 10.1200/JCO.2005.11.007

[23] Hennig G, Gehrmann M, Stropp U, Brauch H, Fritz P, et al (2010) Automated extraction of DNA and RNA from a single formalin-fixed paraffin-embedded tissue section for analysis of both single-nucleotide polymorphisms and mRNA expression. Clin Chem 56: 1845–1853. 20947696 10.1373/clinchem.2010.151233

[24] Altman DG, McShane LM, Sauerbrei W, Taube SE (2012) Reporting recommendations for tumor marker prognostic studies (REMARK): explanation and elaboration. BMC Med 10: 51. 22642691 10.1186/1741-7015-10-51 PMC3362748

[25] Moelans CB, de Weger RA, van Diest PJ (2010) Absence of chromosome 17 polysomy in breast cancer: analysis by CEP17 chromogenic in situ hybridization and multiplex ligation-dependent probe amplification. Breast Cancer Res Treat 120: 1–7. 19760503 10.1007/s10549-009-0539-2

[26] Lambros MB, Simpson PT, Jones C, Natrajan R, Westbury C, et al (2006) Unlocking pathology archives for molecular genetic studies: a reliable method to generate probes for chromogenic and fluorescent in situ hybridization. Lab Invest 86: 398–408. 16446704 10.1038/labinvest.3700390

[27] Sung JS, Park KH, Kim YH (2010) Genomic alterations of chromosome region 11p as predictive marker by array comparative genomic hybridization in lung adenocarcinoma patients. Cancer Genet Cytogenet 198: 27–34. 20303011 10.1016/j.cancergencyto.2009.12.001

[28] Jacquemier J, Spyratos F, Esterni B, Mozziconacci MJ, Antoine M, et al (2013) SISH/CISH or qPCR as alternative techniques to FISH for determination of HER2 amplification status on breast tumors core needle biopsies: a multicenter experience based on 840 cases. BMC Cancer 13: 351. 23875536 10.1186/1471-2407-13-351 PMC3729815

[29] Staaf J, Jonsson G, Ringner M, Vallon-Christersson J, Grabau D, et al (2010) High-resolution genomic and expression analyses of copy number alterations in HER2-amplified breast cancer. Breast Cancer Res 12: R25. 20459607 10.1186/bcr2568 PMC2917012

[30] Jacot W, Fiche M, Zaman K, Wolfer A, Lamy PJ (2013) The HER2 amplicon in breast cancer: Topoisomerase IIA and beyond. Biochim Biophys Acta 1836: 146–157. 23628726 10.1016/j.bbcan.2013.04.004

[31] Kauraniemi P, Kallioniemi A (2006) Activation of multiple cancer-associated genes at the ERBB2 amplicon in breast cancer. Endocr Relat Cancer 13: 39–49. 16601278 10.1677/erc.1.01147

[32] Sahlberg KK, Hongisto V, Edgren H, Makela R, Hellstrom K, et al (2013) The HER2 amplicon includes several genes required for the growth and survival of HER2 positive breast cancer cells. Mol Oncol 7: 392–401. 23253899 10.1016/j.molonc.2012.10.012 PMC5528495

[33] Watari H, Arakane F, Moog-Lutz C, Kallen CB, Tomasetto C, et al (1997) MLN64 contains a domain with homology to the steroidogenic acute regulatory protein (StAR) that stimulates steroidogenesis. Proc Natl Acad Sci U S A 94: 8462–8467. 9237999 10.1073/pnas.94.16.8462 PMC22957

[34] Sahlberg KK, Hongisto V, Edgren H, Makela R, Hellstrom K, et al (2103) The HER2 amplicon includes several genes required for the growth and survival of HER2 positive breast cancer cells. Mol Oncol 7: 392–401. 10.1016/j.molonc.2012.10.012 PMC5528495 23253899

[35] Baumann J, Sevinsky C, Conklin DS (2013) Lipid biology of breast cancer. Biochim Biophys Acta 1831: 1509–1517. 23562840 10.1016/j.bbalip.2013.03.011 PMC3926128

[36] Jornayvaz FR, Lee HY, Jurczak MJ, Alves TC, Guebre-Egziabher F, et al (2012) Thyroid hormone receptor-alpha gene knockout mice are protected from diet-induced hepatic insulin resistance. Endocrinology 153: 583–591. 22147010 10.1210/en.2011-1793 PMC3384074

[37] Fountzilas G, Valavanis C, Kotoula V, Eleftheraki AG, Kalogeras KT, et al (2012) HER2 and TOP2A in high-risk early breast cancer patients treated with adjuvant epirubicin-based dose-dense sequential chemotherapy. J Transl Med 10: 10. 22240029 10.1186/1479-5876-10-10 PMC3275536

[38] Brase JC, Schmidt M, Fischbach T, Sultmann H, Bojar H, et al (2010) ERBB2 and TOP2A in breast cancer: a comprehensive analysis of gene amplification, RNA levels, and protein expression and their influence on prognosis and prediction. Clin Cancer Res 16: 2391–2401. 20371687 10.1158/1078-0432.CCR-09-2471

[39] Murthy SK, Magliocco AM, Demetrick DJ (2005) Copy number analysis of c-erb-B2 (HER-2/neu) and topoisomerase IIalpha genes in breast carcinoma by quantitative real-time polymerase chain reaction using hybridization probes and fluorescence in situ hybridization. Arch Pathol Lab Med 129: 39–46. 15628907 10.5858/2005-129-39-CNAOCN

[40] Glynn RW, Mahon S, Curran C, Callagy G, Miller N, et al (2011) TOP2A amplification in the absence of that of HER-2/neu: toward individualization of chemotherapeutic practice in breast cancer. Oncologist 16: 949–955. 21705665 10.1634/theoncologist.2011-0071 PMC3228145

[41] Zaczek AJ, Markiewicz A, Seroczynska B, Skokowski J, Jaskiewicz J, et al (2012) Prognostic significance of TOP2A gene dosage in HER-2-negative breast cancer. Oncologist 17: 1246–1255. 22871798 10.1634/theoncologist.2012-0023 PMC3481890

[42] Jacobson KK, Morrison LE, Henderson BT, Blondin BA, Wilber KA, et al (2004) Gene copy mapping of the ERBB2/TOP2A region in breast cancer. Genes Chromosomes Cancer 40: 19–31. 15034864 10.1002/gcc.20019

[43] Varga Z, Moelans CB, Zuerrer-Hardi U, Ramach C, Behnke S, et al (2012) Topoisomerase 2A gene amplification in breast cancer. Critical evaluation of different FISH probes. Breast Cancer Res Treat 133: 929–935. 22083232 10.1007/s10549-011-1873-8

[44] Nielsen KV, Muller S, Moller S, Schonau A, Balslev E, et al (2010) Aberrations of ERBB2 and TOP2A genes in breast cancer. Mol Oncol 4: 161–168. 19945923 10.1016/j.molonc.2009.11.001 PMC5527893

[45] Hanna WM, Ruschoff J, Bilous M, Coudry RA, Dowsett M, et al (2014) HER2 in situ hybridization in breast cancer: clinical implications of polysomy 17 and genetic heterogeneity. Mod Pathol 27: 4–18. 10.1038/modpathol.2013.103 23807776

[46] Burrell RA, Juul N, Johnston SR, Reis-Filho JS, Szallasi Z, et al (2010) Targeting chromosomal instability and tumour heterogeneity in HER2-positive breast cancer. J Cell Biochem 111: 782–790. 20665662 10.1002/jcb.22781

[47] Fuchs EM, Kostler WJ, Horvat R, Hudelist G, Kubista E, et al. (2013) High-level ERBB2 gene amplification is associated with a particularly short time-to-metastasis, but results in a high rate of complete response once trastuzumab-based therapy is offered in the metastatic setting. Int J Cancer. 10.1002/ijc.28660 24311197

[48] Montemurro F, Prat A, Rossi V, Valabrega G, Sperinde J, et al (2014) Potential biomarkers of long-term benefit from single-agent trastuzumab or lapatinib in HER2-positive metastatic breast cancer. Mol Oncol 8: 20–26. 24075779 10.1016/j.molonc.2013.08.013 PMC5528507

[49] Smid M, Hoes M, Sieuwerts AM, Sleijfer S, Zhang Y, et al (2011) Patterns and incidence of chromosomal instability and their prognostic relevance in breast cancer subtypes. Breast Cancer Res Treat 128: 23–30. 20632083 10.1007/s10549-010-1026-5

[50] Vincent-Salomon A, Benhamo V, Gravier E, Rigaill G, Gruel N, et al (2013) Genomic instability: a stronger prognostic marker than proliferation for early stage luminal breast carcinomas. PLoS One 8: e76496. 24143191 10.1371/journal.pone.0076496 PMC3797106

[51] Bonnet F, Guedj M, Jones N, Sfar S, Brouste V, et al (2012) An array CGH based genomic instability index (G2I) is predictive of clinical outcome in breast cancer and reveals a subset of tumors without lymph node involvement but with poor prognosis. BMC Med Genomics 5: 54. 23186559 10.1186/1755-8794-5-54 PMC3558323

[52] Roylance R, Endesfelder D, Gorman P, Burrell RA, Sander J, et al (2011) Relationship of extreme chromosomal instability with long-term survival in a retrospective analysis of primary breast cancer. Cancer Epidemiol Biomarkers Prev 20: 2183–2194. 21784954 10.1158/1055-9965.EPI-11-0343 PMC3199437

[53] Iwamoto T, Bianchini G, Booser D, Qi Y, Coutant C, et al (2011) Gene pathways associated with prognosis and chemotherapy sensitivity in molecular subtypes of breast cancer. J Natl Cancer Inst 103: 264–272. 21191116 10.1093/jnci/djq524

[54] Agarwal S, Pappas L, Neumayer L, Kokeny K, Agarwal J (2014) Effect of Breast Conservation Therapy vs Mastectomy on Disease-Specific Survival for Early-Stage Breast Cancer. JAMA Surg. 10.1001/jamasurg.2013.3049 24429935

[55] Munch-Petersen HD, Rasmussen BB, Balslev E (2013) Reliability of histological malignancy grade, ER and HER2 status on core needle biopsy vs surgical specimen in breast cancer. Apmis. 10.1111/apm.12213 24372587

[56] A’Hern RP, Jamal-Hanjani M, Szasz AM, Johnston SR, Reis-Filho JS, et al (2013) Taxane benefit in breast cancer–a role for grade and chromosomal stability. Nat Rev Clin Oncol 10: 357–364. 23648828 10.1038/nrclinonc.2013.67

