# Supplementary material for: Adjusting Breast Cancer Patient Prognosis with Non-HER2-Gene Patterns on Chromosome 17
Source: PLoS One. 2014 Aug 6;9(8):e103707. doi: 10.1371/journal.pone.0103707 (PMC4123879; doi:10.1371/journal.pone.0103707)

**Figure S1: qPCR CN 5-scale classification.** The number of informative samples and copy number (CN) distribution per category is shown. Genes are in lanes, classification for 5’ and 3’ amplicons, and for maximal CN is in rows. Red circles: significant discrepant classification between 5’ and 3’ ends.


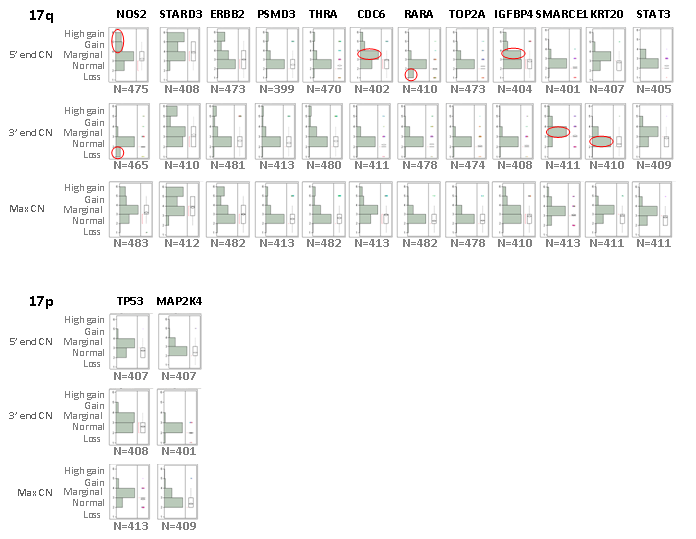

Supplement: Figure S1 — qPCR CN 5-scale classification. The number of informative samples and copy number (CN) distribution per category is shown. Genes are in lanes, classification for 5′ and 3′ amplicons, and for maximal CN is in rows. Red circles: significant discrepant classification between 5′ and 3′ ends. (DOC) [file pone.0103707.s001.doc]
