# Supplementary material for: Adjusting Breast Cancer Patient Prognosis with Non-HER2-Gene Patterns on Chromosome 17
Source: PLoS One. 2014 Aug 6;9(8):e103707. doi: 10.1371/journal.pone.0103707 (PMC4123879; doi:10.1371/journal.pone.0103707)

**Figure S2: CN value distribution upon logarithmic transformation (natural logarithm).** **a,** 5’ end; **b,** 3’ end; **c,** highest value of the two. CN ranged between 2 – 4 in the majority of tumors, indicating low gains and/or DNA replication in the majority of cells


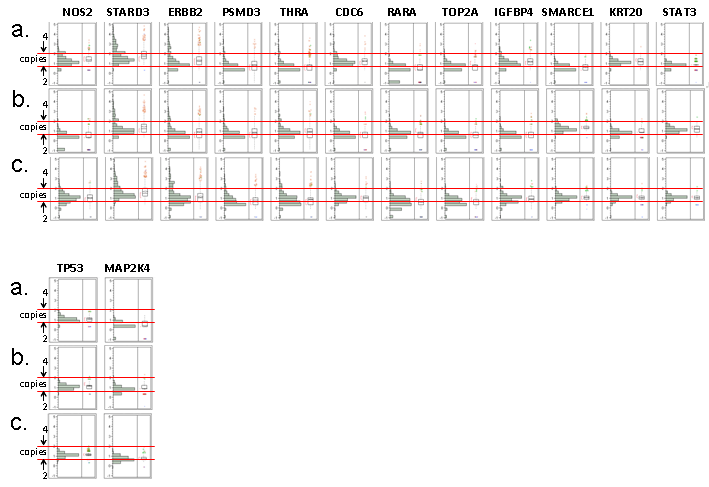

Supplement: Figure S2 — CN value distribution upon logarithmic transformation (natural logarithm). a, 5′ end; b, 3′ end; c, highest value of the two. CN ranged between 2–4 in the majority of tumors, indicating low gains and/or DNA replication in the majority of cells. (DOC) [file pone.0103707.s002.doc]
