# Supplementary material for: Adjusting Breast Cancer Patient Prognosis with Non-HER2-Gene Patterns on Chromosome 17
Source: PLoS One. 2014 Aug 6;9(8):e103707. doi: 10.1371/journal.pone.0103707 (PMC4123879; doi:10.1371/journal.pone.0103707)

**Figure S3: Clustered correlations for 5’ and 3’ end copy number (CN) values and for maximal CN.** **A,** log transformed; **B,** 5-scale classification. **C,** maximal CN (5-scale) Similar to log transformed CN in Figure 1, core HER2 amplicon genes (STARD3, ERBB2, PSMD3, THRA) cluster together for both amplicons per gene and for maximal CN. CN for NOS2, RARA and IGFBP4 also correlate to each other, while TOP2A status is only vaguely related to these genes.


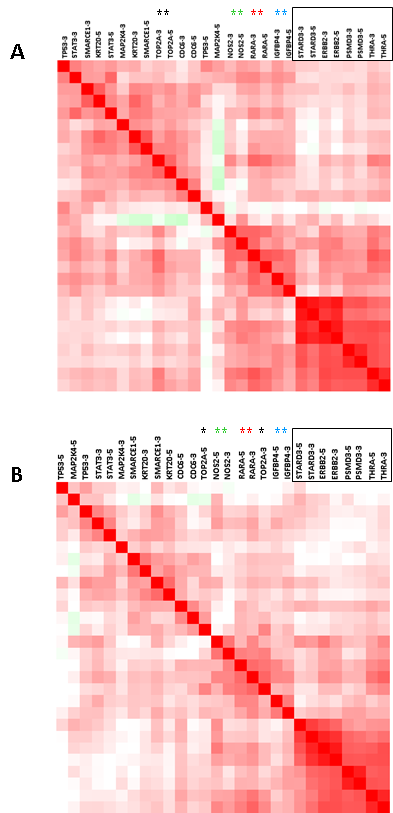


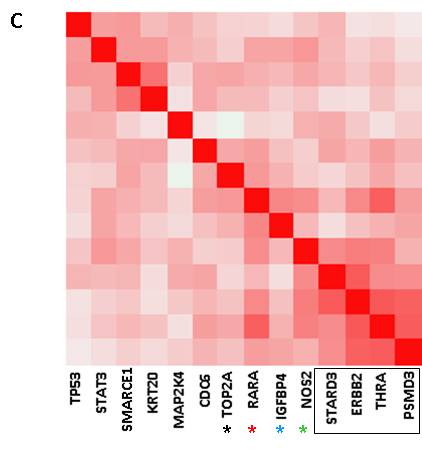

Supplement: Figure S3 — Clustered correlations for 5′ and 3′ end copy number (CN) values and for maximal CN. A, log transformed; B, 5-scale classification. C, maximal CN (5-scale) Similar to log transformed CN in Figure 1, core HER2 amplicon genes (STARD3, ERBB2, PSMD3, THRA) cluster together for both amplicons per gene and for maximal CN. CN for NOS2, RARA and IGFBP4 also correlate to each other, while TOP2A status is only vaguely related to these genes. (DOC) [file pone.0103707.s003.doc]
